# Polycentric Exoprosthetic Knee Joints – Extent of Shortening During Swing Phase

**DOI:** 10.33137/cpoj.v3i1.33768

**Published:** 2020-07-29

**Authors:** T.M. Köhler, M Bellmann, S Blumentritt

**Affiliations:** 1 Clinical Research and Services, Research Biomechanics, Ottobock SE & Co. KGaA, Göttingen, Germany.; 2 Private University of Applied Science, Göttingen, Germany.

**Keywords:** Amputation, Ground Clearance, Knee Joint, Polycentric, Prosthesis, Limb Loss, Rehabilitation

## Abstract

**BACKGROUND:**

An often assumed advantage of polycentric knee joints compared to monocentric ones is the improved ground clearance during swing phase due to the geometric shortening of the lower leg segment (LLS).

**OBJECTIVE:**

To investigate whether polycentric knee joints considerably improve ground clearance and to evaluate the influence of prosthetic alignment on the extent of ground clearance.

**METHODOLOGY:**

11 polycentric and 2 monocentric knee joints were attached to a rigid, stationary testing device. Shortening of the LLS and the resulting ground clearance during knee flexion were measured. Prosthetic components were mounted at the same height and the anterior-posterior position was in accordance with the manufacturer's alignment recommendations.

**FINDINGS:**

Shortening of up to 14.7 (SD=0.0) mm at the instance of minimal ground clearance during swing phase was measured. One knee joint elongated by 4.4 (SD=0.0) mm. Measurements of the ground clearance demonstrated differences up to 25.4 (SD=0.0) mm. One monocentric knee joint provided more ground clearance when compared to 8 of the polycentric knee joints investigated.

**CONCLUSION:**

Only some polycentric knee joints shorten appreciably during swing phase. With an optimized prosthetic alignment and a well-designed swing phase control, a monocentric knee joint may generate greater ground clearance compared to a polycentric knee joint.

## INTRODUCTION

Tripping is a safety risk for amputees and it is mainly affected by ground clearance during swing phase. In mid– swing, the toe is at the minimum distance to the ground while the shank rotates forward.^1,2^ To compensate for a lack of ground clearance, transfemoral amputees often perform compensatory movements including plantar flexion of the intact ankle during prosthetic swing (vaulting), lifting the hip on the prosthetic side during prosthetic swing (hip hiking), and swinging the prosthesis forward in an arc by abducting the hip early in swing and then adducting the hip late in swing (circumduction). These gait abnormalities reduce walking efficiency and are therefore undesirable.^1-4^ During prosthetic alignment, the length of the prosthesis can be reduced when compared to the intact limb and this might result in increased ground clearance during walking. However, this intervention can lead to other compensatory movements,^5^ reduced walking efficiency^6^ and lower back pain.^7^ Therefore, a prosthesis that provides a technical solution for ensuring adequate ground clearance during swing phase on the prosthetic side is desirable. Sockets with vacuum suspension can minimize longitudinal movement (pistoning) between the socket and limb^8^ and thus reduce the functional elongation of the prosthesis. Prosthetic feet generating ankle dorsiflexion during swing phase showed more ground clearance compared to conventional energy storing and returning feet.^9,10^ Polycentric knee joints also showed more ground clearance during swing phase when compared to monocentric knee joints.^1,11^

Due to their technical design, prosthetic knee components can be divided into two main categories: monocentric and polycentric knee joints. Monocentric knee joints have a single center of rotation (CR) that is independent of the knee flexion angle. In polycentric knee joints, the upper and lower parts of the knee joint are usually connected via a four-bar linkage mechanism. They rotate around an instantaneous center of rotation (ICR), which is dependent upon the knee flexion angle. The ICR results from the intersection of the longitudinal axes of the anterior and the posterior linkages. Depending on the specific linkage mechanism, the ICR is usually located outside of the knee joint construction itself.^12^ Consequently, there is a high degree of stance phase safety provided at heel strike since a fully extended knee joint results in an ICR that is located posterior to the knee joint and this is posterior to the vector of the ground reaction force. For patients with knee disarticulation or long transfemoral residual limbs, polycentric knee joints are preferred from a cosmetic point of view when seated, due to the minimized protrusion at the distal end of the socket in a flexed knee position.^11^ As an additional advantage, the shortening of the shank during swing phase or virtual ankle dorsiflexion is often stated.^1, 11, 13^

Due to their design, polycentric knee joints are able to generate greater ground clearance in a flexed knee position compared to monocentric ones based on this shortening effect. Hence, in a study from 1996, ground clearance values were 9-32 mm higher for polycentric joints.^11^ Another study reported an average of 22 mm more ground clearance for the investigated polycentric knee joints.^1^ Currently, there is a larger variation in the length and orientation of the linkages in polycentric knee joints than in the formerly reported studies. Consequently, for the knee joints investigated in this study shortening effects of less than the formerly reported 9 mm were expected.

### Objective

The aim of this study was to investigate whether polycentric knee joints generally show a marked shortening of the lower leg segment during prosthetic swing phase resulting in a clear advantage for patients regarding tripping. Furthermore, the influence of prosthetic alignment with regards to ground clearance was also examined.

## METHODOLOGY

### Knee joints

**Figure 1:**
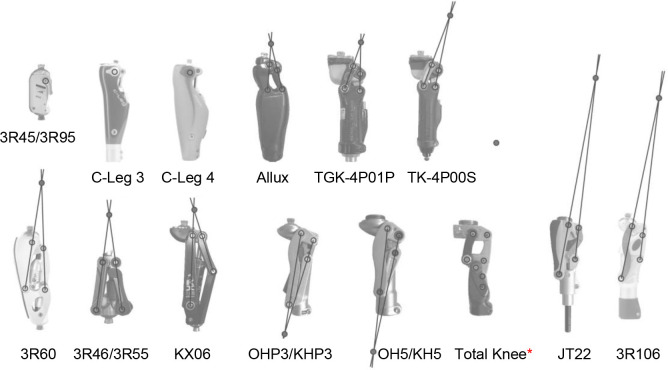
Polycentric and monocentric knee joints investigated with schematic illustration of the ICR respectively (scaling is not uniform between the knee joints), *according to Van de Veen PG, 2001.^14^

In this study, 11 polycentric knee joints were investigated: The 3R46/3R55, 3R60, 3R106 (**Ottobock** SE & Co. KGaA, Duderstadt, Germany), Total Knee 2000, OHP3/KHP3, OH5/KH5 (**Össur**, Reykjavik, Iceland), TGK-4P01P, TK-4P00S (**Teh Lin**, Taipei, Taiwan), JT22 (**Uniprox**, Zeulenroda, Germany), KX06 (**Blatchford**, Basingstoke, United Kingdom) and Allux (**Nabtesco**, Tokyo, Japan). The monocentric knee joints 3R45/3R95 and C-Leg (Ottobock SE & Co. KGaA, Duderstadt, Germany) were also examined. Except for the 3R60 and Total Knee 2000, each of the polycentric knee joints utilizes a 4-bar linkage system. The fifth axis of the 3R60 only affects stance phase. Therefore, the 3R60 is effectively a 4-bar linkage knee joint during swing phase. The Total Knee uses a 7 axis linkage mechanism and all of the axes are involved during swing phase motion (Figure 1).

### Prosthetic alignment

**Figure 2:**
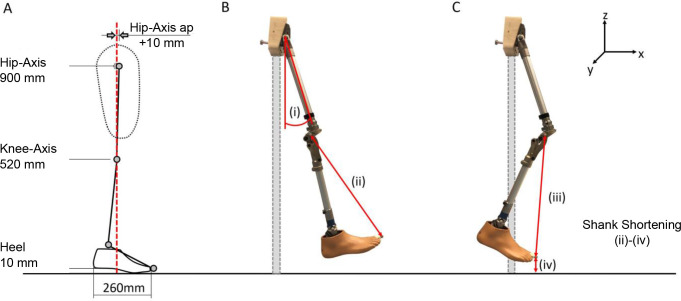
Schematic illustration of: (A) Prosthetic alignment. (B) Experimental setup with knee joint in full extension, (i) hip flexion angle (individual tube inclination was considered), (ii) distance between knee (extended) and big toe marker. (C) Experimental setup when knee joint is flexed, (iii) distance between knee (flexed) and big toe marker, (iv) minimum ground clearance.

Bench alignment of the prosthesis was performed using a L.A.S.A.R assembly (Ottobock SE & Co. KGaA, Duderstadt, Germany). For each alignment, a Trias foot (Ottobock SE & Co. KGaA, Duderstadt, Germany) with a length of 260 mm and an effective heel height of 10 mm was used. The 7E7 (Otto Bock, Duderstadt, Germany) was utilized as a hip joint. The knee joint was adjusted to a height of 520 mm and the 7E7 to 900 mm. The hip joint axis was located 10 mm anterior to the reference line since the physiological hip center of rotation is located approximately 10 mm anterior to the greater trochanter and this is typically used as the reference point for the prosthetic socket anterior-posterior position (Figure 2). The length of the prosthesis was approximated according to an average person (male, 1.80 m) based on anthropomorphic data.

The A-P (anterior-posterior) position of each knee component and foot were aligned according to the manufacturer’s instructions for the individual knee joint (Table 1). Following these recommendations, the alignments should provide realistic data for each specific knee joint investigated in this study. The Allux and C-Leg were tested according to their recommended alignment. Subsequently, these knee joints were re-tested with the others respective recommended alignment to observe the effect of alignment differences on ground clearance without the possible influence of shortening effects.

**Table 1:** Alignment position of prosthetic knee joints investigated.

Knee joint	Position foot	Position alignment reference point* [mm] anterior + /posterior -
Allux	FP 1 / FP 2	0/0
KX06	FP 1	0
Total Knee	FP 2	+11**
OHP3/KHP3	FP 2	0
OH5/KH5	FP 2	0
TGK-4P01P	FP 2	0
TK-4P00S	FP 2	0
JT22	FP 1	-25
3R46/3R55	FP 1	-10
3R60	FP 1	0
3R106	FP 1	0
3R45/3R95	FP 1	-15
C-Leg	FP 1 / FP 1 / FP 2	+5 / 0 / 0

*Upper anterior axis for polycentric knee joints, knee rotation axis for monocentric knee joints **Offset between reference axis defined by the manufacturer (aligned on the alignment reference line) and upper anterior axis is approximately 11 mm.

Each foot was positioned as follows:

- Foot Position 1 (FP 1): The middle of the foot is positioned 30 mm anterior to the alignment reference line
- Foot Position 2 (FP 2): The alignment reference line divides the foot into 1/3 rear foot and 2/3 forefoot

In order to ensure that the motion only occurred in the sagittal plane, the axes of rotation for the hip and knee as well as the distal surface of the foot were all aligned perpendicular to the sagittal plane for all test setups.

### Experimental setup

**Figure 3:**
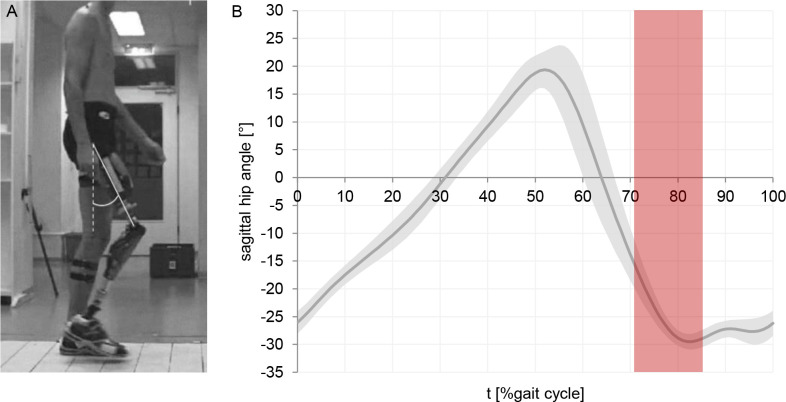
(A) Transfemoral amputee during level ground walking (example- the patient has given written informed consent for use of the picture). (B) Typical mean sagittal hip angle of transfemoral amputees while walking on level ground (n=6).^17^ Red area indicates assumed range where minimum ground clearance occurs.

The test prosthesis was connected via the proximal part of the 7E7 joint to a stationary device. This configuration allowed a step-less adjustment of the hip flexion angle. The position of the hip axis of rotation was stationary. The study from Winter cites an average hip angle of 23° at the instance of minimal ground clearance while walking at a selfselected, medium walking speed.^15^ In this study, four hip flexion angles were evaluated: 15°, 20°, 25° and 30° (angle from vertical). This range should cover the potential hip flexion of a transfemoral amputee at the instance of minimum ground clearance during swing phase (Figure 3). Due to different pyramid adapter positions for each knee joint, the resulting inclination of the tube adapter between hip and knee joint varies during bench alignment. Starting from the individual inclinations of the tube adapter, the hip joint was flexed by the respective angles investigated. This ensures identical effective hip flexion angles for all investigated knee joints (Figure 2).

### Measuring system

Kinematic parameters were measured via an optoelectronic 12-camera motion capture system at a sampling rate of 200 Hz (Vicon Bonita B10, Vicon Peak, Oxford, United Kingdom). Four retro-reflective markers were attached to the prosthesis: Hip joint axis, knee joint axis (Polycentric: upper anterior axis), lateral ankle adapter screw, big toe (hallux) (Figure 2). 3D trajectories (X, Y, Z) of the markers were captured with an accuracy of 0.5 mm.^16^

### Experimental procedure

The specifically defined hip angle was adjusted prior to each measurement. The knee joint was flexed and extended manually from the fully extended position to a knee flexion angle of approximately 90° and then back into full extension six times during the measurement (six motion cycles).

### Data analysis

Minimum ground clearance was defined as the event when the z-component of the big toe marker reached its minimum. Shortening of the lower leg segment was defined by the difference of the distance between the knee marker and the big toe marker at full knee extension, and at the instant of minimal ground clearance. The distances were calculated within the sagittal plane, via the XZ- coordinates (Figure 2). This definition was used because it considers the possible virtual ankle dorsiflexion that was previously reported.^1, 11^ All results are described as mean values over six motion cycles.

### Validation of the measurement method

Due to minimal changes in the prosthetic alignment, setup alignment and marker placement, deviations in the investigated parameters might occur. To validate the reproducibility of the measurement method, the previously described procedure (1. prosthetic alignment, 2. experimental setup and application of reflective marker, 3. experimental procedure) was repeated 3 times (including 15 motion cycles each) for one representative hip flexion position (25°) involving the 3R60 and C-Leg.

#### Evaluation of the inter-test reliability:

For each repetition, the mean was calculated for lower leg shortening and minimum ground clearance. An ANOVA based reliability measure was calculated with a tolerance threshold of SD=1.5 mm, given the following formula:

- Variance explained by threshold, assuming an equal distribution of means $\bar{x_{l}}$ over the range of their average $\bar{x}\pm 1.5mm:{\text{ Var }}_{\text{threshold }}=\frac{1}{12}(1.5-(-1.5){)}^{2}=0.75$
- Reliability $=\min\left[\left[1;1-\frac{{\text{ Var }}_{\text{between }}}{{\text{ Var }}_{\text{total }}}+\frac{{\text{ Var }}_{\text{threshold }}}{{\text{ Var }}_{\text{total }}}\right]\right]$ with *Var*_*total*_ the total variance and *Var*_*between*_ the variance of the means.

Subsequent ranges of the 3 mean values were calculated respectively:

- minimum ground clearance: 1.6 mm (3R60) and 0.6 mm (C-Leg)
- lower leg shortening: 2.2 mm (3R60) and 0.3 mm (C-Leg)

With a maximum range of 2.2 mm measured, the authors evaluate the measurement reliability as adequate for the statements made in this study (reliability coefficients 0.85-1).

#### Evaluation of the intra-test reliability:

Given the data from 15 motion cycles, the range and precision (half-length of the confidence interval for the mean) was calculated for each repetition, assuming that the point estimations of mean and variance were based on only 6 measurements. This assumption allowed an estimation of the expected precision when using only 6 motion cycles instead of 15. The resulting maximum range (r) and minimum precision (pr) were:

- minimum ground clearance: r = 0.4 mm, pr = 0.13 mm (3R60); r = 0.5 mm, pr = 0.13 mm (C-Leg)
- lower leg shortening: r = 0.6 mm, pr = 0.15 mm(3R60); r = 0.4 mm, pr = 0.13 mm (C-leg)

The evaluation of the intra-test reliability showed that the rigid stationary device offers a constant motion of the prosthesis (deviation for measured parameters approximately SD=0.3 mm). Thus, mean values over six motion cycles seem to provide sufficient precision (0.06-0.15 mm).

## RESULTS

### Lower leg shortening

**Figure 4:**
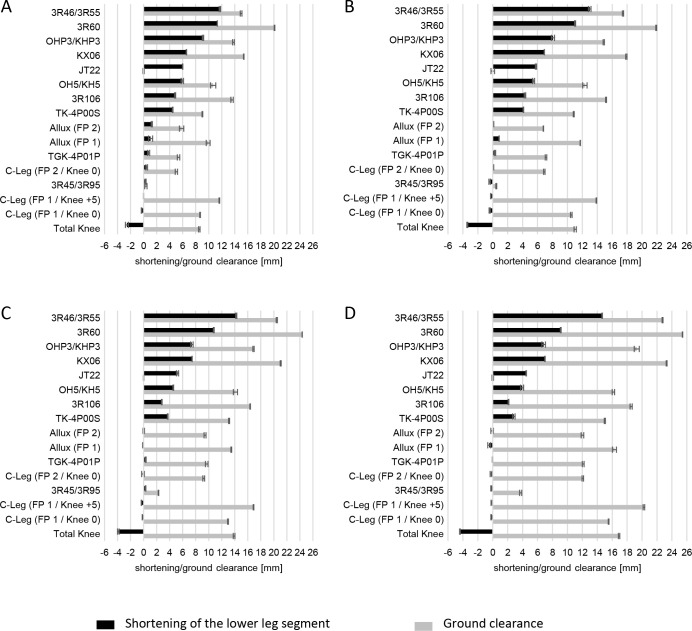
Shortening of the lower leg segment (black) and ground clearance (grey) at the instance of minimal ground clearance (A) 15°, (B) 20°, (C) 25° and (D) 30° hip flexion; mean with standard deviation.

Except for the Total Knee and Allux, each polycentric knee joint generated a shortening of the lower leg segment during minimal ground clearance for all hip angles investigated. Shortening of up to 14.7 (SD=0.0) mm was measured for the 3R46/3R55. The Total Knee elongated over all investigated hip angles with a maximum of 4.4 (SD=0.0) mm at 30° hip flexion. The Allux knee joint elongated (max. 0.5 mm) at a hip flexion angle of 25° and 30°. The shortening tends to decrease with increasing hip flexion angles for all joints expect for the KX06 and 3R46/3R55 (Figure 4).

### Minimum ground clearance

When comparing the results of ground clearances, the minimum value measured at each hip flexion angle was set to 0 mm. The lowest ground clearance was identified with the JT22 for all hip flexion angles. In comparison, the 3R60 provides up to 25.4 (SD=0.0) mm greater ground clearance. At 15° hip flexion, 6 polycentric knee joints showed lower ground clearance than the C-Leg (12 mm with FP 1 / Knee +5). At 25° and 30° hip flexion, 8 polycentric knee joints demonstrated lower ground clearance than this monocentric knee joint (Figure 4).

### Comparison of different alignment methods

A comparison of different alignment methods with regards to toe clearance was conducted for the Allux and C-Leg. At 15° hip flexion, the Allux showed 4.1 mm greater ground clearance with the FP 1 than with the FP 2 and 4.3 mm greater ground clearance at 30° hip flexion, respectively. With the C-Leg, ground clearance at 15° hip flexion was 6.6 mm greater with the FP 1 / Knee +5 than with the FP 2 / Knee 0 and 8.2 mm greater at 30° hip flexion, respectively (Figure 4).

## DISCUSSION

The aim of this study was to investigate whether polycentric knee joints provide a substantial shortening of the lower leg segment during swing phase. The results indicate that this is not valid for each polycentric design. Due to the individual length and orientation of the linkages of each polycentric knee joint investigated a large variation of shortening effects were observed. As mentioned by Anand et al.^18^, a higher ICR seems to result in higher ground clearance.^19^ This should be considered in the development of the geometric design of a polycentric knee joint.^18^ However, this correlation could not be observed. Polycentric knee joints with a more proximally located ICR (Figure 1: Total Knee, JT22, 3R106) demonstrate lower leg shortening of up to 6.0 (SD=0.0 mm) or even elongation of up to 4.4 (SD=0.0) mm. The 3 polycentric knee joints demonstrating the greatest shortening of the lower leg segment (Figure 1: KX06, 3R60, 3R46/3R55) share other similarities (Figure 3, Table 2): The posterior linkage is relatively long and tilted anteriorly. The ICR is (compared to the Total Knee, JT22, 3R106) more distally and more anteriorly located (close to the longitudinal axis of the knee joint). These knee joints reached 7.0 (SD=0.0) mm up to 14.7 (SD=0.0) mm lower leg shortening.

**Table 2:** Evaluation of the geometric design of the polycentric knee joints investigated based on Figure 1 (visual comparison of proportions).

Knee joint	ICR height*	ICR A-P position	Length anterior linkage	Length posterior linkage	Tilt anterior linkage	Tilt posterior linkage	LLS (25°) [mm]
Total Knee	high	posterior	short	short	backward	backward	-3.9 (SD=0.1)
Allux (FP 2)	low	centered	short	short	backward	forward	0.0 (SD=0.0)
TGK-4P01P	low	posterior	short	short	backward	backward	0.3 (SD=0.1)
3R106	high	posterior	long	short	backward	backward	2.8 (SD=0.0)
TK-4P00S	medium	posterior	short	short	backward	backward	3.7 (SD=0.0)
OH5/KH5	below knee joint	anterior	long	short	backward	backward	4.6 (SD=0.1)
JT22	high	posterior	medium	short	backward	backward	5.2 (SD=0.2)
OHP3/KHP3	below knee joint	anterior	long	short	backward	backward	7.4 (SD=0.2)
KX06	low	centered	long	medium	backward	forward	7.4 (SD=0.0)
3R60	medium	centered	long	medium	backward	forward	10.8 (SD=0.1)
3R46/3R55	medium	centered	long	long	backward	forward	14.2 (SD=0.1)

*high-low: ICR above knee joint; high: more proximally located, low: more distally located. Abbreviations: ICR instantaneous center of rotation, A-P anterior posterior, LLS lower leg shortening

Furthermore, the impact of the prosthetic alignment on ground clearance was considerable. Thus, some of the knee joints generated relatively minor ground clearance even though they shortened to a large extent of up to 14.7 (SD=0.0) mm.

The a-p position of the knee joint caused by the alignment affects the ground clearance in two ways: The more posterior the knee joint, the

**Figure 5:**
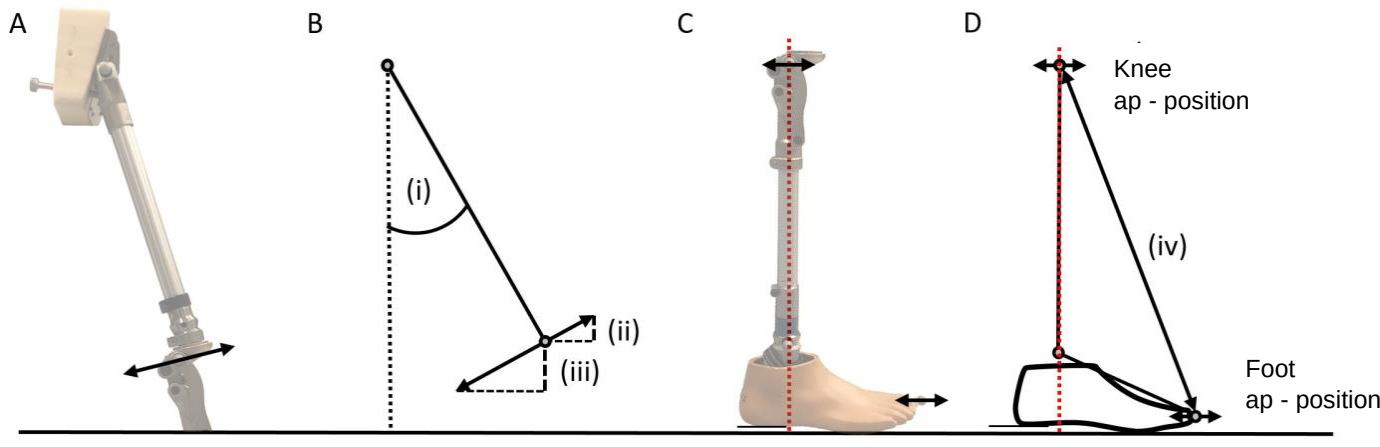
Effect of a-p positioning of the knee and foot components (A) experimental set up, (B) schematic diagram of the effect of a-p positioning of the knee, (i) hip flexion angle, (ii) gain of ground clearance, (iii) loss of ground clearance, (C) prosthetic alignment, (D) schematic diagram of shortening and elongation of the shank, respectively, (iv) depending on the position of the knee and foot.

1. closer the knee center of rotation is to the ground during hip flexion. This effect increases with increasing hip flexion angles due to the more distally located trajectory of the knee reference point (Figure 5 A-B).
2. greater the distance between the knee center of rotation and the big toe and this results in a longer prosthesis at the instance of minimal ground clearance (Figure 5 C-D).

This substantive alignment effect can be clearly seen with the 3R95 monocentric knee joint which is aligned 20 mm more posteriorly than the monocentric C-Leg (FP 1 / Knee +5) and this results in 16.5 mm less ground clearance at 30° hip flexion.

Thus, ground clearance during swing phase is enhanced when the knee joint is placed in a more anterior position. As an example, one monocentric knee joint (C-Leg, FP 1 / Knee +5) generated greater ground clearance then up to 8 polycentric knee joints due to its specific alignment depending on hip flexion angle.

Additionally, the effect of the foot position was investigated and resulted in the following observation:

The more anterior the position of the foot, the longer the forefoot and the longer the distance between the knee center of rotation and the big toe. Hence, ground clearance decreases during swing with a more anteriorly positioned foot (Figure 5 C-D). As a conclusion, the FP 1 exhibited a considerable advantage over the FP 2 for the two knee joints that were investigated with these two alignment methods (C-Leg, Allux).

In this study the extent of geometric shortening of the lower leg segment with different polycentric knee joints, as well as the impact of prosthetic alignment on the resulting ground clearance was investigated. Another potential aspect regarding the resulting ground clearance, not being investigated in this study, is the property of the swing phase control of the prosthetic knee joint. Based on the investigation of Winter, small differences in knee flexion angle considerably affect ground clearance. 1.4° difference in the knee flexion angle resulted in a difference of 4.5 mm ground clearance, seen in non-amputees.^15^ Depending on the individual swing phase control (appropriate adjustment of the swing flexion and extension resistance) of the prosthetic knee joint, the knee angle progression during swing phase can vary significantly.^20,21^ Thus, the swing phase control might have an influence on the resulting ground clearance. Therefore, further experiments with the knee joints presented in this study are suggested to clarify this additional aspect.

### Limitations

The prosthetic and experimental setup as well as the marker positioning was adjusted with the greatest care. However, minimal deviations might occur, as seen in the validation of the measurement method. Due to these effects, non-realistic shortenings of the shank were measured even for monocentric knee joints (e.g. C-Leg 4, (SD=0.4) mm). The authors assume an overall accuracy of SD=1.1 mm for the investigated parameters. This assumption is based on the validation of the entire measurement method. Nevertheless, this accuracy is sufficient for the statements that were made in this study.

## CONCLUSION

In summary, not all polycentric knee joints shorten appreciably at the instant when a stumble might occur. Thus, the previously stated functional advantage of greater ground clearance for patients must be reconsidered. A slightly more anterior position of the knee joint or a more posterior position of the foot can compensate for or even exceed the extent of the geometric shortening of the shank of some polycentric knee joints.

## DECLARATION OF CONFLICTING INTERESTS

Mr. Thomas Maximilian Köhler and Dr. Malte Bellmann are employees of Ottobock SE & Co. KGaA.

## AUTHOR CONTRIBUTION

Each of the authors concurs with the content in the final manuscript.

- **Mr. Thomas Maximilian Köhler, MSc:** study design, execution of the gait lab measurements, data analysis and interpretation, drafting of the article
- **Dr. Malte Bellmann:** study design, execution of the gait lab measurements, data analysis and interpretation, drafting of the article.
- **Prof. Dr. Siegmar Blumentritt:** drafting of the article.

## SOURCES OF SUPPORT

This research received no specific grant from any agency in the public, commercial or not-for-profit sectors.
